# Cell Surface Protein Disulfide Isomerase Regulates Natriuretic Peptide Generation of Cyclic Guanosine Monophosphate

**DOI:** 10.1371/journal.pone.0112986

**Published:** 2014-11-24

**Authors:** Shuchong Pan, Horng H. Chen, Cristina Correia, Haiming Dai, Tyra A. Witt, Laurel S. Kleppe, John C. Burnett, Robert D. Simari

**Affiliations:** 1 Division of Cardiovascular Diseases, Mayo Clinic, Rochester, MN, United States of America; 2 Division of Oncology Research, Mayo Clinic, Rochester, MN, United States of America; Maastricht University, Netherlands

## Abstract

**Rationale:**

The family of natriuretic peptides (NPs), including atrial natriuretic peptide (ANP), B-type natriuretic peptide (BNP), and C-type natriuretic peptide (CNP), exert important and diverse actions for cardiovascular and renal homeostasis. The autocrine and paracrine functions of the NPs are primarily mediated through the cellular membrane bound guanylyl cyclase-linked receptors GC-A (NPR-A) and GC-B (NPR-B). As the ligands and receptors each contain disulfide bonds, a regulatory role for the cell surface protein disulfide isomerase (PDI) was investigated.

**Objective:**

We utilized complementary *in vitro* and *in vivo* models to determine the potential role of PDI in regulating the ability of the NPs to generate its second messenger, cyclic guanosine monophosphate.

**Methods and Results:**

Inhibition of PDI attenuated the ability of ANP, BNP and CNP to generate cGMP in human mesangial cells (HMCs), human umbilical vein endothelial cells (HUVECs), and human aortic smooth muscle cells (HASMCs), each of which were shown to express PDI. In LLC-PK1 cells, where PDI expression was undetectable by immunoblotting, PDI inhibition had a minimal effect on cGMP generation. Addition of PDI to cultured LLC-PK1 cells increased intracellular cGMP generation mediated by ANP. Inhibition of PDI *in vivo* attenuated NP-mediated generation of cGMP by ANP. Surface Plasmon Resonance demonstrated modest and differential binding of the natriuretic peptides with immobilized PDI in a cell free system. However, PDI was shown to co-localize on the surface of cells with GC-A and GC-B by co-immunoprecpitation and immunohistochemistry.

**Conclusion:**

These data demonstrate for the first time that cell surface PDI expression and function regulate the capacity of natriuretic peptides to generate cGMP through interaction with their receptors.

## Introduction

Members of the natriuretic peptide (NP) family, atrial natriuretic peptide (ANP), B-type natriuretic peptide (BNP), and C-type natriuretic peptide (CNP) are central regulators of sodium and water balance, blood volume, and arterial pressure as well as myocardial and vascular structure and function. [1] The actions of the NPs are mediated through the guanylyl cyclase (GC)-associated receptors GC-A and GC-B. [2] Both receptors contain three different functional domains, an extracellular ligand-binding domain, a transmembrane domain and an intracellular domain which consists of a kinase homology domain and a GC domain. GC-A and GC-B exist on cell surfaces as homodimers or homotetramers. [3], [4], [5] When ligands bind to the ligand-binding pocket of the dimer, the conformation of receptors is changed and signaling through the transmembrane domain results in ATP binding. These steps are essential for generation of the second-messenger, cGMP. [6], [7], [8] ANP and BNP preferentially bind and activate GC-A while CNP predominantly binds and activates GC-B, and through generation of cGMP, have been used as therapeutics for cardiorenal disease. [9], [10]

Protein disulfide isomerase (PDI) is a multifunctional cytoplasmic and membrane-bound enzyme with known chaperone activity. [11], [12] PDI has dithiol-disulfide oxidoreductase activities which can reduce, oxidize, and isomerize disulfide bonds. Previous studies have identified PDI at the plasma membrane of lymphocytes, platelets, endothelial cells, hepatocytes, and some cancer cells. [11], [12], [13] Membrane-bound PDI can catalyze reduction of disulfide bonds in cell surface proteins and augment cell adhesion and migration. [14], [15], [16] PDI has also been shown to regulate viral entry into cells by altering the conformation of viral fusion proteins and cell surface receptors. [17], [18] PDI also interacts with membrane proteins, such as platelet surface protein β3 integrin, [19] CD4, and CXCR4, on the surface of T cells. [20], [21] Thus, PDI has been identified as an important enzyme which may act on disulfide bonds and regulate peptide signaling. The current studies investigate for the first time, the role of PDI as a novel regulator of natriuretic peptide activity specially in the regulation of cGMP generation.

## Methods

### Reagents

ANP, BNP, and CNP were purchased from Phoenix Pharmaceuticals Inc. Anti-PDI monoclonal antibody RL90 was purchased from Novus Biologicals (Littleton, CO). Bacitracin and purified PDI were purchased from Sigma (Saint Louis, MO) and Novaplus (New York, NY). PDI siRNA was purchased from Santa Cruz Biotechnology, Inc (Santa Cruz, CA).

### Cell culture

Human umbilical vein endothelial cells (HUVECs), human aortic smooth muscle cells (HASMCs), and pig kidney epithelial cells (LLC-PK1) were purchased from American Type Culture Collection (ATCC) (Manassas, VA). HUVECs were cultured in endothelial growth medium (EGM-2) with supplements (Lonza, Hopkinton, MA). HASMCs were cultured in smooth muscle cells growth medium (SmBM) with supplements (Lonza). Primary human glomerular mesangial cells (HMCs) (Cell Systems Inc., Kirkland, WA) were grown in mesangial growth media with supplements (Cell Systems Inc.). LLC-PK1 cells were grown in Dullbecco's Modification of Eagle's Medium (DMEM) containing 10% FBS. All cell cultures were grown at 37°C in humidified 5% CO_2_ air. Primary cells from passage 3 to 7 were used in this study.

### Mice

10- to 16-week-old female C57BL/6 mice were obtained from Jackson Laboratory. All animal experiments complied with the standards stated in the *Guide for the Care and Use of Laboratory Animals* (Institute of Laboratory Animal Resources, National Academy of Sciences, Bethesda, MD) and were approved by the Mayo Clinic Institutional Animal Care and Use Committee.

### Measurements of total cellular cGMP from cells in culture

Total cellular cGMP was measured using the BIOTRAK cGMP enzyme immunoassay system Kit (GE Healthcare) as previously described. [22] Briefly, cells were plated in 96-well plates and incubated overnight to reach 80% confluence. The cells were stimulated with different concentrations of synthetic natriuretic peptides dissolved in basic culture medium containing 0.5 mM IBMX for 30 minutes, then lysed. All samples were completed in triplicate for each experiment. The amount of cGMP was calculated from a standard curve that was generated in parallel. Bacitracin (an inhibitor of PDI), RL90 (a PDI-specific antibody), and PDI were used to investigate the effect of PDI on cGMP generation.

### siRNA transfection

HMCs were seeded in 6-well plates at 1×10^5^ cells/cm2 with growth medium and incubated overnight. The next day, 8 µl of siRNA or scrambled oligomers (10 µM) were diluted in 100 µl siRNA transfection medium, and 6 µl of the siRNA transfection agent were diluted into 100 µl siRNA transfection medium in separate tubes. The two mixtures were combined, mixed gently and incubated at room temperature for 45 minutes. 800 µl siRNA transfection medium was added to each complex (200 µl) and mixed well. 70% confluent cells were rinsed with siRNA transfection medium and immediately overlaid with the transfection mixture. Cells were incubated for 6 hours, after which 1 ml fresh complete growth medium was added to each well. Cells were grown for an additional 24 hours and then used for analysis of protein expression of PDI or for cGMP stimulation experiments.

### Diphtheria Toxin sensitivity assay

PDI-dependent diphtheria toxin (DT) sensitivity of the cells was determined using a modified protocol of the Promega (Madison, WI) Cell Titer 96 Aqueous One Cell Proliferation Assay as previously described. [25] Briefly, cells were plated in 96-well plates at 5×10^3^ cells per well and incubated overnight. Cells were washed once with Hank's Balanced Salt Solution (HBSS) and then incubated with DT (Sigma, MS) for 30 minutes at 37°C. Cells were washed 3 times with HBSS and incubated with growth medium in a CO_2_ incubator for 72 hours. Cell viability was measured using a Promega Kit according to the manufacturer's instructions.

### Preparation of cell lysates and membrane fractions

Whole cell extracts were prepared with CHAPS Lysis and Immunoprecipitation Buffer (FIVEphoton Biochemicals, San Diego, CA). Membrane fractions of HMCs were prepared with the ProteoExtract Native Membrane Protein Extraction kit (Calbiochem, San Diego, CA) according to the manufacturer's instructions. Protein concentrations of cell lysates and membrane fractions were determined by protein assay (Bio-Rad). Samples were aliquoted and kept at −80°C until use.

### Western blotting

Conventional western blotting was used to identify PDI in cellular proteins and was described previously. [22]

### Affinity Measurements by Surface Plasmon Resonance (SPR)

SPR measurements were performed at 25°C on a Biacore T200 biosensor (GE Healthcare). PDI full-length protein (Sigma, St. Louis, MO) was immobilized onto a CM5 sensorchip using an amine coupling immobilization kit (GE Healthcare) as instructed by the supplier. After the chip was washed with Biacore running buffer (150 mM NaCl, and 0.005% (w/v) Polysorbate 20, 10 mM HEPES, pH 7.4), peptides ANP, BNP or CNP were injected at 40 ul/min for 30 s and allowed to dissociate for 150 s. Residual bound peptide was desorbed with 350 mM NaCl. Binding kinetics were derived from sensorgrams using Bioevaluation software version 4.1.1 (GE Healthcare).

### *In vivo* PDI inhibition

Blood samples were collected from each mouse using retro-orbital bleeding as baselines before experiments. Mice were infused via tail vein with ANP (0.04 ug/g(BW)) 5 minutes after administration of the PDI-specific antibody RL90 (4 ug/g(BW)). Blood samples were obtained at 5 minutes from alternate eyes. cGMP concentration in plasma samples was measured as described above.

### Immunoprecipitation

Membrane fractions from HMCs were solubilized with CHAPS Immunoprecipitation Buffer (FIVEphoton Biochemicals) and immunoprecipitated according to manufacturer's instruction. Briefly, 500 µl of the solubilized membrane fraction containing 300 µg of protein was incubated with 1 µg of anti-GC-A, anti-GC-B antibodies or rabbit IgG for 4 hours to overnight at 4°C. The immune complexes were trapped on protein A/G Plus-Agarose (Santa Cruz Biotechnology), washed three times with PBS, then boiled in sample loading buffer for 5 minutes. The samples were centrifuged to pellet the agarose, and the supernatants were subjected to SDS-PAGE and western blotted with anti-PDI monoclonal antibody.

### Confocal immunofluorescence microscopy

Colocalization of PDI and GC-A in HUVECs, and PDI and GC-B in HMCs was assessed by immunofluorescence. Cells were grown in growth medium in 8-well chamber slides overnight, washed with PBS and fixed with methanol at -20°C. HUVECs were blocked with 10% normal goat serum before incubating with rabbit anti-GC-A serum 1∶100 (Abcam, Cambridge, MA) and mouse anti-PDI Ab 1∶300 (Novus, Littleton CO) cocktail. GC-A was visualized using goat anti-rabbit Ab AF 488 1∶500 (Molecular Probes, Eugene, OR), and PDI was visualized using goat anti-mouse AF 594 (Molecular Probes). HMCs were blocked with 10% normal goat serum before incubating with rabbit anti-PDI Ab 1∶100 (Enzo Life Science, Farmingdale NY) and mouse anti-GC-B Ab 1∶1000 (Abcam, Cambridge, MA) cocktail. PDI was visualized using goat anti-rabbit Ab AF 488 1∶500 (Molecular Probes, Eugene, OR), and GC-B was visualized using goat anti-mouse AF 594 (Molecular Probes). Cell nuclei were stained with Hoechst (Sigma). Confocal microscopy was performed with a Zeiss LSM 510 system (Zeiss, Germany). Two lasers were used: an argon laser (488 nm) for the FITC conjugate and a helium neon laser (543 nm) for the rhodamine-conjugate. There was no overlap in fluorescence emission between these probes.

### Statistical Analysis

Data are expressed as the mean+SEM. Statistical analyses were performed using Student's *t* test for *in vitro* studies and one-way ANOVA for *in vivo* experiments. *p*<0.05 was considered significant.

## Results

### PDI regulation of NP-mediated cGMP stimulation *in vitro*

As the NPs are potent stimulators of cGMP in vascular and renal cells, we sought to determine whether PDI expression and activity modulate stimulation of cGMP by NPs. We defined the role of PDI on NP-mediated cGMP stimulation in cells using bacitracin (an inhibitor of PDI and other thiol isomerases in the PDI family), PDI siRNA and an anti-PDI monoclonal antibody, RL90. [17], [18], [19]

First, we verified that bacitracin inhibited PDI function, showing it inhibited bacitracin-dependent DT sensitivity in HMCs (**Figure S1**). Then, to exclude the effects of protease contamination from bacitracin, [23] we determined that bacitracin did not affect the expression of CG-Bin HMCs (**Figure S2)**, and that it did not lose its inhibitory effects on cGMP levels in the presence of protease inhibitors (**Figure S3**).

**Figure 1:**
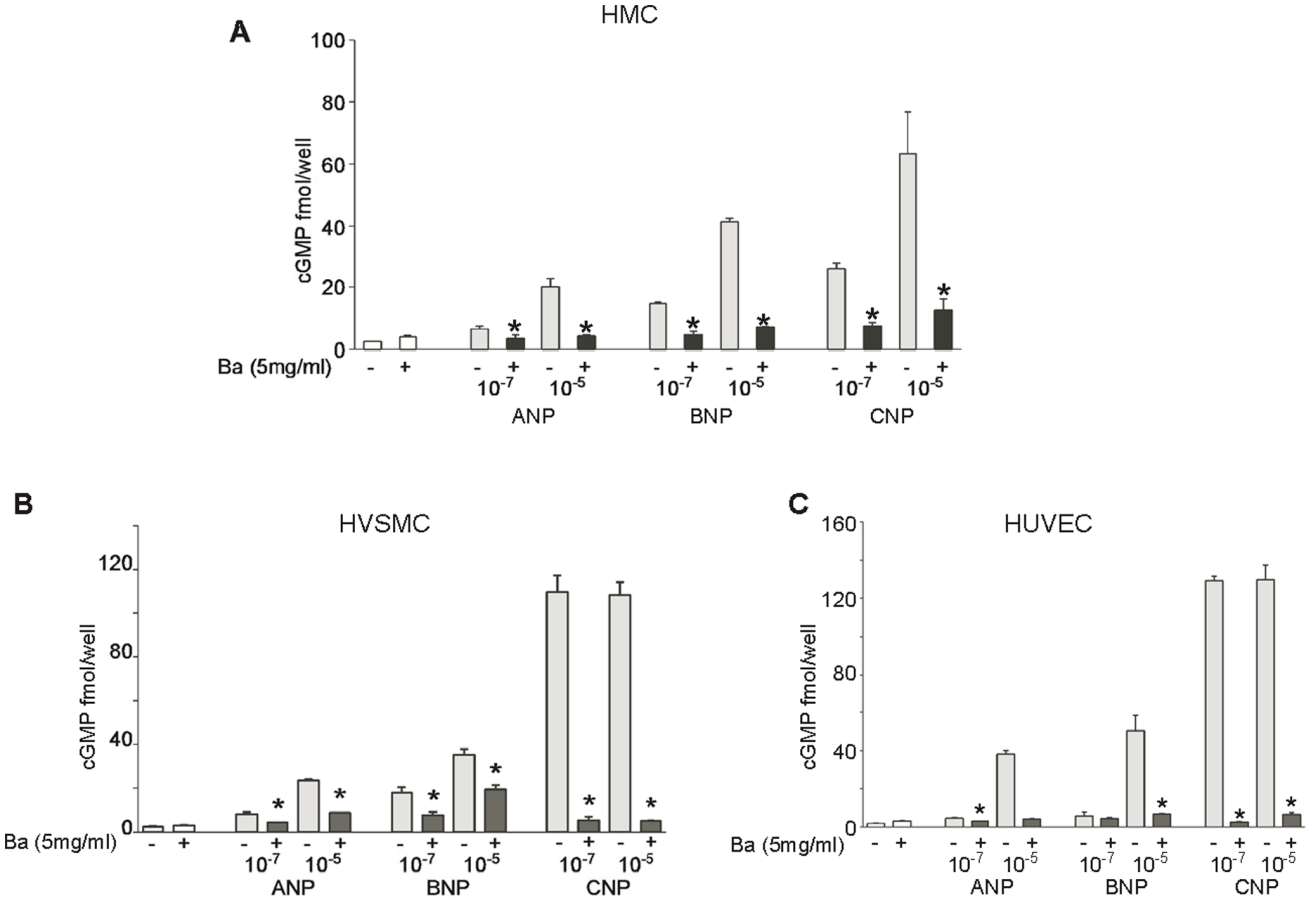
Bacitracin inhibited PDI attenuates NP-mediated generation of cGMP in vascular cells. Bacitracin (Ba) inhibits NPs activation in HMC (A), HVSMC (B) and HUVEC (C) * p ≤0.05 vs samples without bacitracin addition. (Error bars, +SD from 3 independent experiments, samples were triplicated in each experiment).

We then tested the ability of bacitracin to inhibit cGMP generation by ANP, BNP, and CNP in HMCs(A), HASMCs(B) and HUVECs(C), which express both GC-A and GC-B, but GC-B in greater abundance. [24], [25] ANP, BNP, and CNP-mediated increases in cGMP levels were significantly attenuated when bacitracin was added (Fig. 1). The inhibitory effects of bacitracin were dose dependent (data not shown).

**Figure 2:**
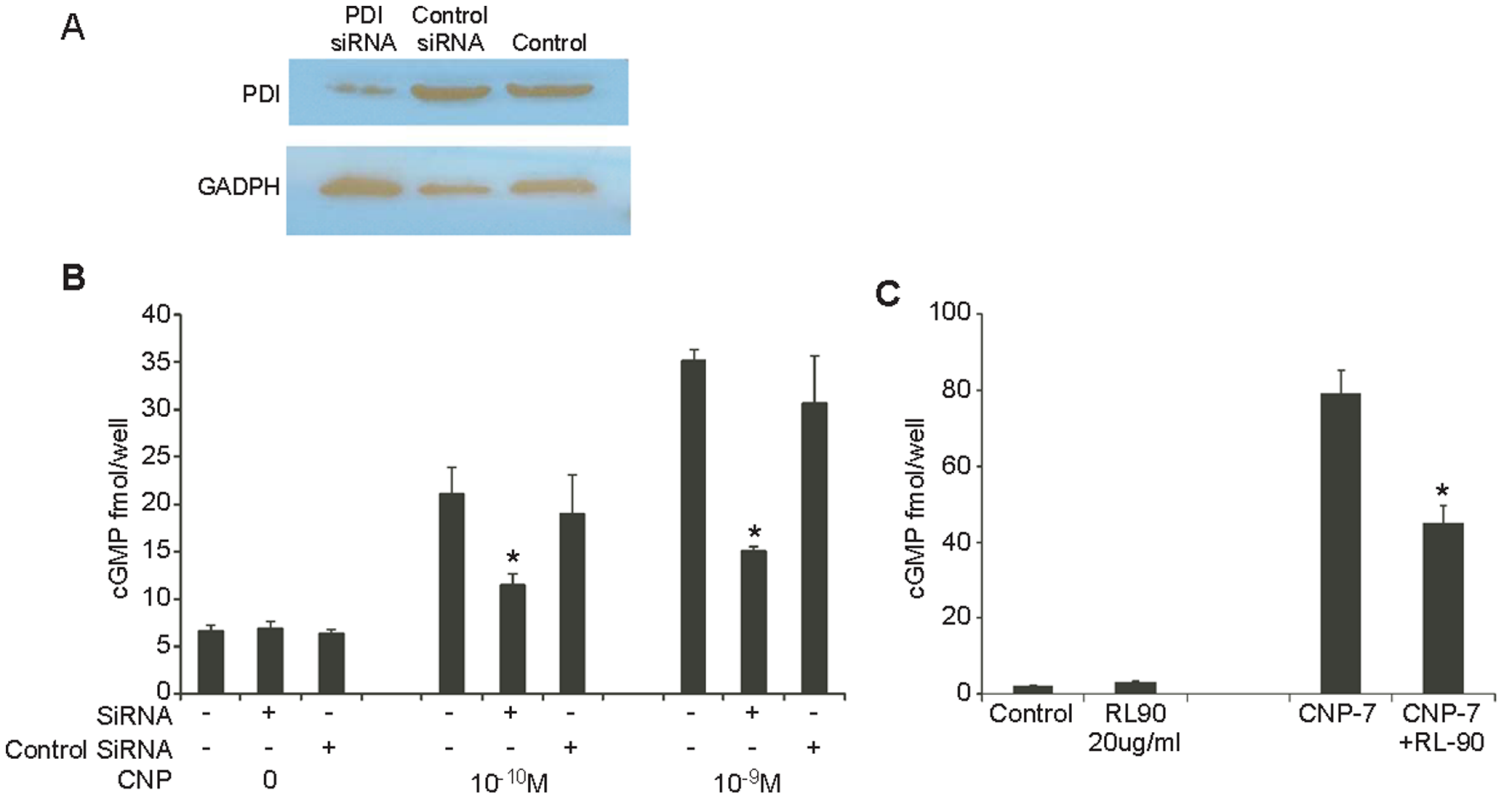
Down regulated PDI attenuates CNP-mediated cGMP activation in HMCs. **A**. Western blotting with RL90 antibody shows partial knockdown of PDI in HMCs. **B**. Effect of PDI knockdown on cGMP activation. Data are expressed as mean+SEM vs control cells without PDI siRNA treatment (*P≤0.05). **C**. PDI inhibition by RL90 decreases ANP, BNP and CNP-mediated cGMP generation in HMC (*p≤0.05). (Error bars, +SD from 3 independent experiments, samples were triplicated in each experiment).

To assess a more specific inhibition of PDI, we used small interfering RNA (siRNA) to silence PDI expression. Partial knockdown of PDI expression and activity was achieved in HMCs after siRNA treatment as determined by immunoblot (Fig. 2A) and by DT sensitivity assay. (Fig. S4). The siRNA treated cells were then subjected to CNP stimulation. Knockdown of PDI expression decreased CNP stimulation of cGMP approximately 50% compared with control siRNA-treated cells (Fig. 2B).

Finally, we used RL90, an anti-PDI specific antibody, to inhibit PDI activity in CNP treated HMCs. CNP mediated cGMP production was reduced approximately 43% with 20 µg/ml RL90 compared to control (Fig. 2C). Similar results were seen with ANP and BNP stimulation. These data suggest that inhibition of cell membrane bound PDI activity down-regulates cGMP stimulation by natriuretic peptides. Taken together, these studies suggest that in cells that express PDI, its inhibition by bacitracin, siRNA, or inhibitory antibodies attenuates natriuretic peptide cGMP generation in vitro.

**Figure 3:**
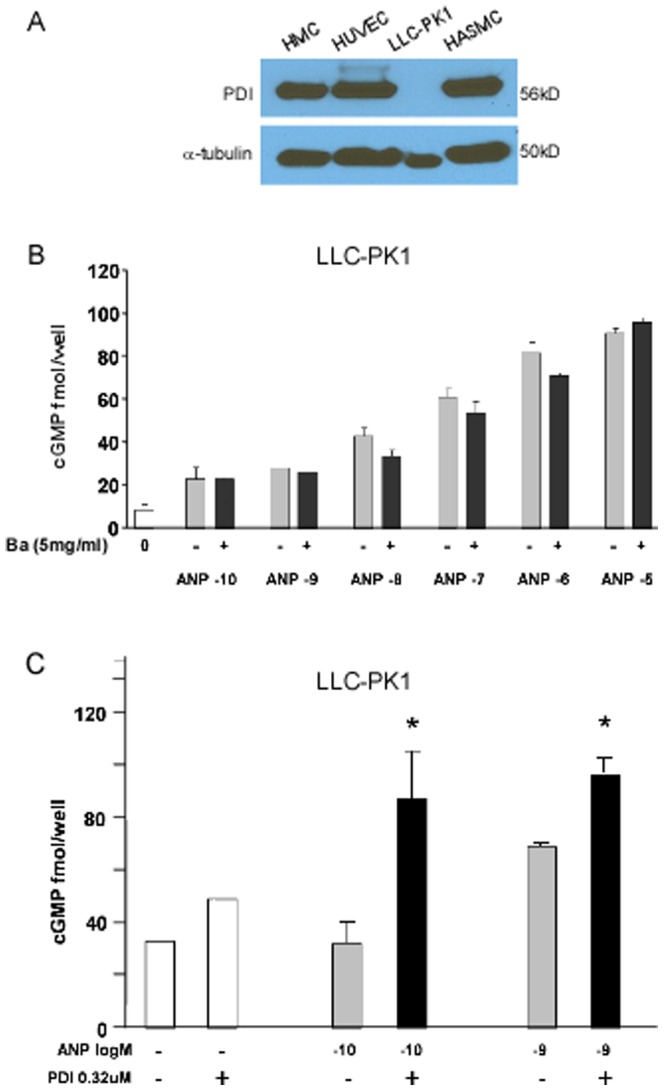
PDI effects on cGMP generation of LLC-PK1 cells stimulated by ANP. **A**. PDI expression in vascular smooth muscle cells (VSMC), endothelial cells (HUVEC), and mesangial cells (HMC). Expression of PDI was not detected on LLC-PK1 cells (porcine renal tubular cells). **B**. Bacitracin had no effect on cGMP generation of LLP-CK1 cells stimulated by ANP. **C**. Addition of purified PDI increased cGMP production significantly in LLC-PK1 cells compared to the cells without PDI. Data are expressed as mean+SEM vs control without PDI addition (*p value ≤0.05). (Error bars, +SD from 3 independent experiments, samples were triplicated in each experiment).

In contrast, in LLC-PK1 cells which predominantly express GC-A, but lack detectable PDI expression (Fig.3A), bacitracin did not inhibit ANP-mediated increases in cGMP (*p>0.05) (Fig.3B). Addition of purified PDI to LLC-PK1 cells increased ANP stimulated cGMP production by 159% and 39% compared with the cells without PDI (Fig. 3C).

### PDI regulation of NP-mediated cGMP stimulation *in vivo*

**Figure 4:**
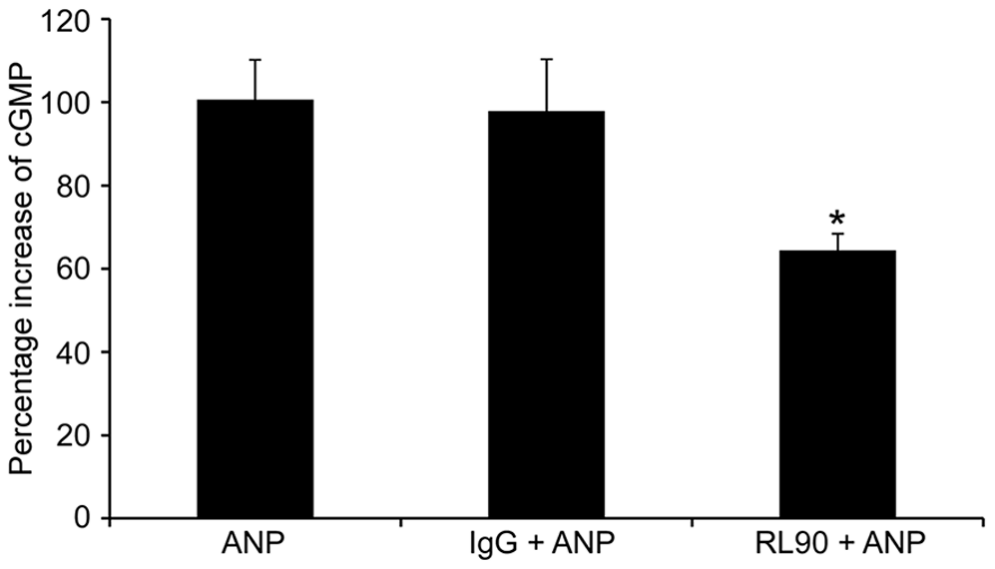
PDI inhibition attenuates NP-mediated generation of cGMP in vivo. Mice were given ANP with or without PDI inhibitors. Blood samples were collected, cGMP levels assessed. Data are expressed as mean+SEM (n = 6 mice). * p value ≤0.05 vs ANP with addition of RL90 antibody, and ** p value ≤0.001 with addition of Bacitracin.

To determine whether PDI inhibits natriuretic peptides function *in vivo*, the PDI inhibitor RL90 was infused into mice prior to administration of ANP. ANP infusion increased cGMP plasma concentrations, which was decreased 32% by addition of RL90 treatment(Fig. 4). As baseline cGMP concentration varied between mice, we expressed our data as percent change of cGMP. These data suggest that PDI inhibition attenuated cGMP stimulation by NPs *in vivo*.

### Direct PDI binding to NPs

**Figure 5:**
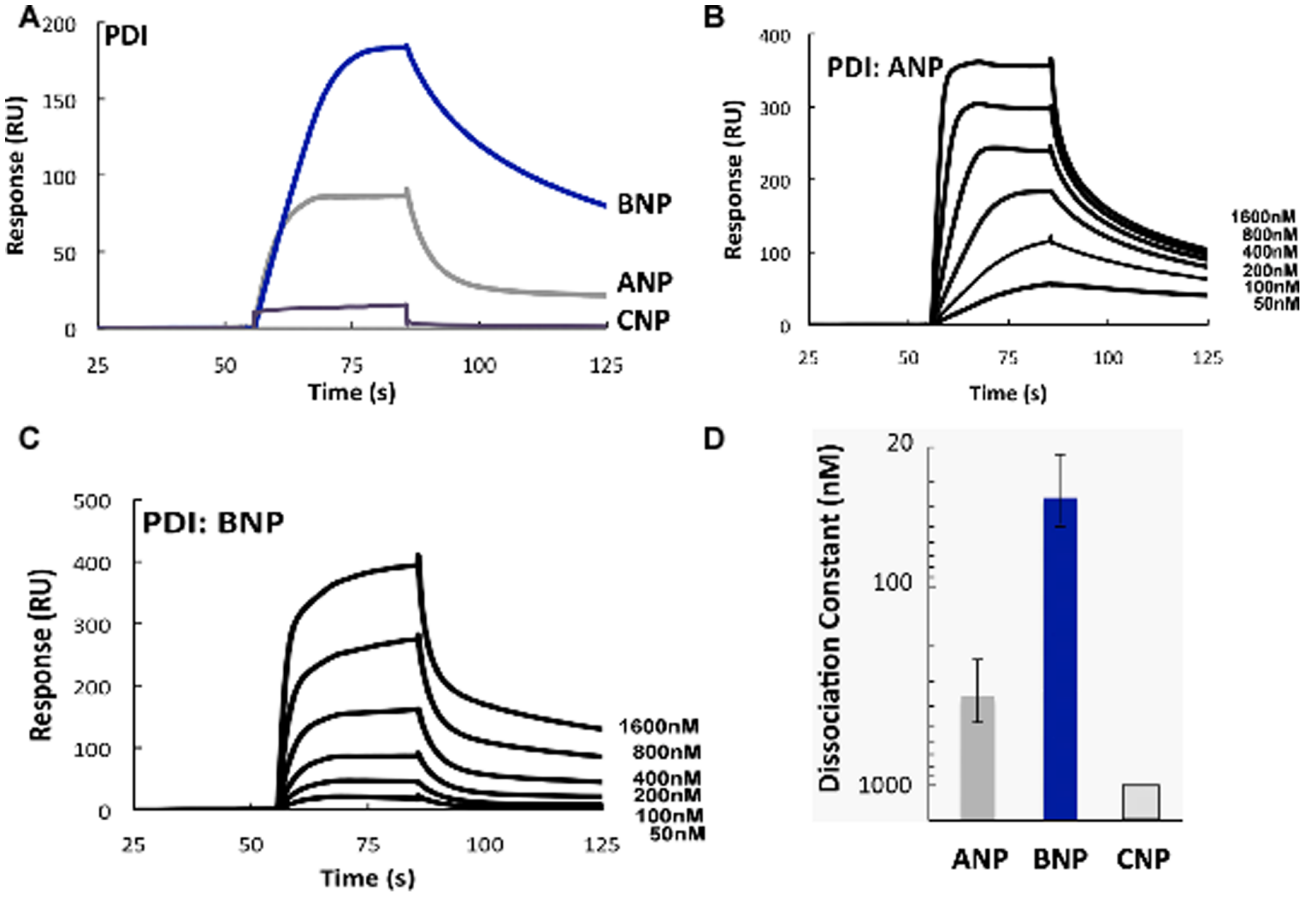
Differential binding of NPs and PDI. **A**. Binding between immobilized PDI and 200 nM NPs was analyzed by SPR. **B and C.** Surface plasmon resonance (SPR) signals when immobilized PDI protein was exposed to varying concentrations (0–1600 nM) of ANP and BNP (representative from 3 independent experiments). **D**. Dissociation constants for complexes of NPs with PDI protein. (Error bars, +SD of 3 independent experiments using different chips and peptide preparations.).

To determine whether the effect of PDI can be correlated with direct binding of NPs to PDI, we assessed their interactions with immobilized PDI protein using Surface Plasma Resonance (SPR) (Fig. 5A). ANP showed moderate binding with PDI (Fig. 2B) while BNP had the highest binding affinity with PDI and displayed dose-dependent increases in binding. (Fig 5C). The mean equilibrium dissociation constant for ANP was 125 nM, compared to 35 nM for BNP. CNP displayed much lower affinity (Fig. 5D). Thus, while there is evidence to support direct binding in vitro, there is variability among the NPs which is not consistent with the uniform effects of PDI on NP function.

### PDI expression and colocalization with GC-A and GC-B

**Figure 6:**
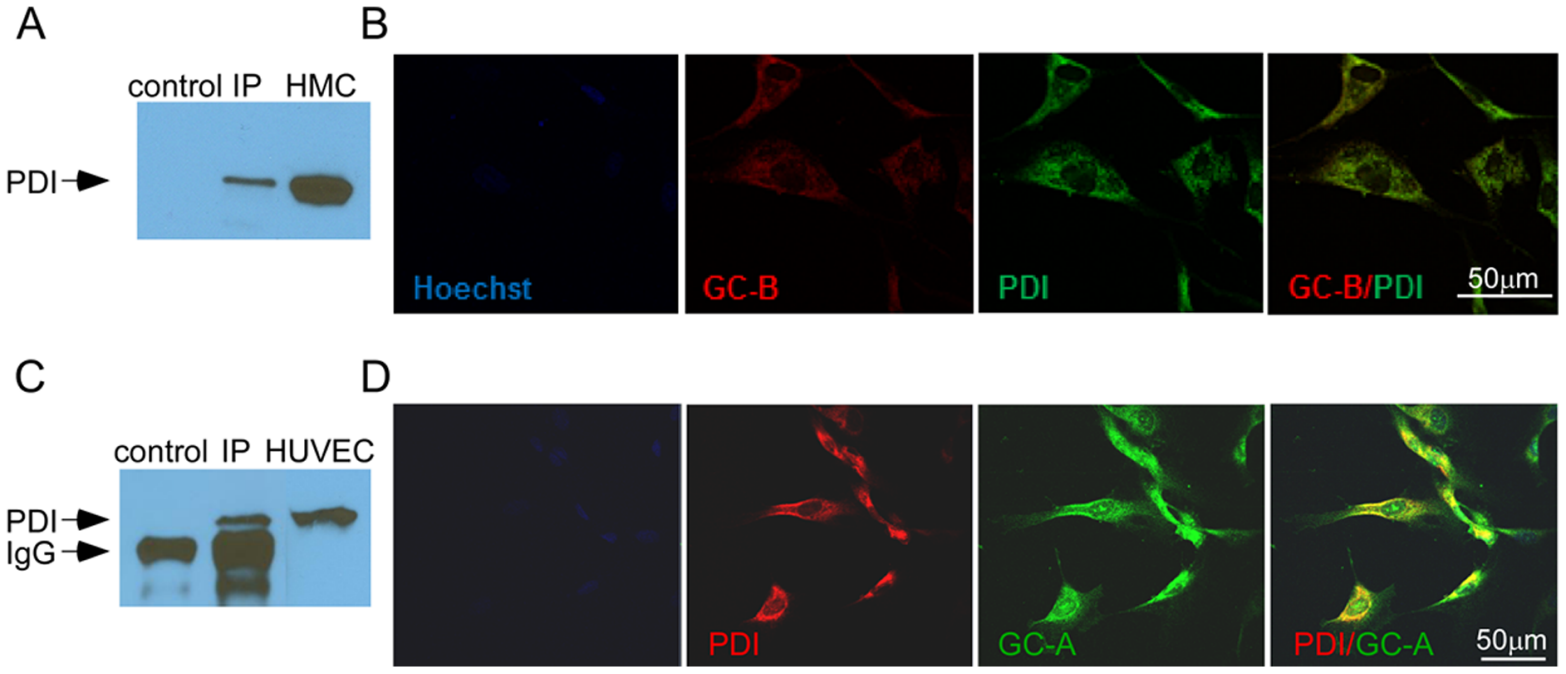
Cell surface PDI associated with GC-A and GC-B. **A. and C.** Proteins were pulled down with anti GC-A or anti GC-B antibodies from HUVEC or HMC membrane preparations and detected with anti-PDI antibody (RL90)compared to control IP (no anti GC-A or anti GC-B antibody) and non-immunoprecipitated proteins from HMC and HUVEC. **B**. Co-localization of PDI (red) and GC-A (green) in HUVEC. **D**. Co-localization of PDI (green) and GC-B (red) in HMC.

We then sought to determine whether PDI co-localized with GC-A or GC-B. Results from immunoprecipitation showed that membrane bound PDI, detected in HUVECs and HMCs, also co-precipitated using anti-GC-A and anti-GC-B antibody respectively, but is not present in a mouse IgG control (Fig. 6A, C). Confocal microscopy demonstrated that PDI and GC-A and GC-B co-localize in HUVECs and HMCs (Fig. 6B, D) but not in LLC-PK1 cells (data not shown). Taken together, these studies suggest that PDI co-localizes with GC-A and GC-B in HUVEC and HMC respectively and suggest that PDI regulation of natriuretic peptide-mediated cGMP generation may be mediated through its effects on GC-A and GC-B.

## Discussion

We present here for the first time, that inhibition of PDI activity attenuates NP-mediated generation of the NP second messenger cGMP. PDI binds NPs differentially, and co-localizes with GC-A, GC-B. These results suggest that membrane-bound PDI physically interacts with natriuretic peptides (ANP and BNP) and their receptors (GC-A and GC-B) and is an important allosteric modulation of this cGMP system.

In the current studies, cGMP generation by NPs in HMCs, HASMCs, and HUVECs was decreased significantly (up to ∼90%) when PDI activity was inhibited by bacitracin, RL90, or siRNA-mediated knockdown. The greater effect seen with bacitracin compared to RL90 or siRNA knockdown is likely due to its broader range of inhibitory effects on other thiol isomerases. In contrast, LLC-PK1 cells, which lack detectable PDI expression, displayed no significant change of cGMP generation with bacitracin. Moreover, addition of purified PDI to LLC-PK1 cells increased the ability of NP to stimulate cGMP, suggesting that PDI activity, although modulatory of NP function, is not required in NP stimulated cGMP. In addition, by utilizing multiple cell types that express different levels of GC-A and GC-B. and by immunoprecipitation and immunostaining of HMCs and HUVECs, we demonstrate that PDI has similar effects on CG-A and GC-B, revealing PDI may modulate the cGMP action of all NPs (i.c. ANP, BNP and CNP).

PDI has an essential role in the oxidative folding and chaperone-mediated quality control of proteins in secretory pathways. [26] Previous studies have demonstrated that PDI is involved in a wide range of physiological and disease processes. [27] Toldo and colleagues identified PDI as a cardioprotective factor in cardiomyocyte ischemia due to its ability to relieve ER stress and prevent accumulation of unfolded proteins. [28] Beside its function within the ER, PDI also has been demonstrated to catalyzes the isomerization of disulfide bonds of cell surface proteins. [29], [30], [31], [32] The interaction changes the protein conformation of cell surface proteins resulting in the alternation of their functions.

The important role of the disulfide bonds in the natriuretic peptide receptors is well established. The extracellular domain (ECD) (peptide binding domain) of GC-A contains six cysteines which form three disulfide bonds. [33] The binding of ANP to the ECD affects GC-A homodimer quaternary structure causing the two juxtamembrane domains in the dimer to translate in opposite directions. [34] Misono et al have proposed that this ligand-induced rotation mechanism in the juxtamembrane region triggers transmembrane signal transduction. [35] These studies indicate that the quaternary structure of ECD is critical for the function (ligand binding and activation) of natriuretic peptide receptors. The internal cysteines of the ECD formed disulfide bonds are the main forces to maintain and alter the molecular structure of the receptors.

As such, we would speculate that the conformation of these critical domains is modulated by thiol isomerases including cell surface PDI which would explain our experimental findings. Our data suggest that PDI co-localizes with the NP receptors (GC-A and GC-B) and is capable of binding to NP ligands (such as ANP, BNP and CNP) allowing for a modulation of its effects. The nature of this relationship and its stoichiometry is beyond the scope of this current paper and will be a focus of future studies. However, our data suggest that this modulatory role of PDI is not limited to any given NP ligand or GC-A or GC-B alone but is more generalizable. These findings could aid in further understanding the modulation of NP action in cardiovascular and renal diseases in which alterations in PDI activity could impact NP activation of cGMP.

## Supporting Information

Figure S1 A. Bacitracin inhibits PDI-dependent DT toxicity. HMCs were treated with DT with or without bacitracin. Bacitracin increased cell survival significantly, demonstrating the ability to inhibit PDI-dependent cell toxicity (*p<0.05). (Error bars, +SD from 3 independent experiments).
(TIF)

Figure S2 Bacitracin treatment does not affect GC-B expression on HMC. Western blot analysis of HMCs with and without Bacitracin treatment. There was no difference in GC-B expression with exposure to Ba.
(TIF)

Figure S3 Protease inhibition does not change affect of bacitracin on cGMP generation by NPs in HMC. cGMP levels of ANP and CNP stimulated HMCs with and without bacitracin(Ba). Ba was added with or without protease inhibitor cocktail (according to the manufacturer instruction (Error bars, +SD from 3 independent experiments)).
(TIF)

Figure S4 Knockdown of PDI expression by siRNA in HMC inhibits PDI-dependent DT toxicity. DT sensitivity analysis shows siRNA knockdown PDI expression in HMC results in less cell surface PDI and increase cell survival 3+ fold (*p<0.001). (Error bars, +SD from 3 independent experiments).
(TIF)
