# Supplementary figures and images for: Cell Surface Protein Disulfide Isomerase Regulates Natriuretic Peptide Generation of Cyclic Guanosine Monophosphate
Source: PLoS One. 2014 Nov 24;9(11):e112986. doi: 10.1371/journal.pone.0112986 (PMC4242536; doi:10.1371/journal.pone.0112986)

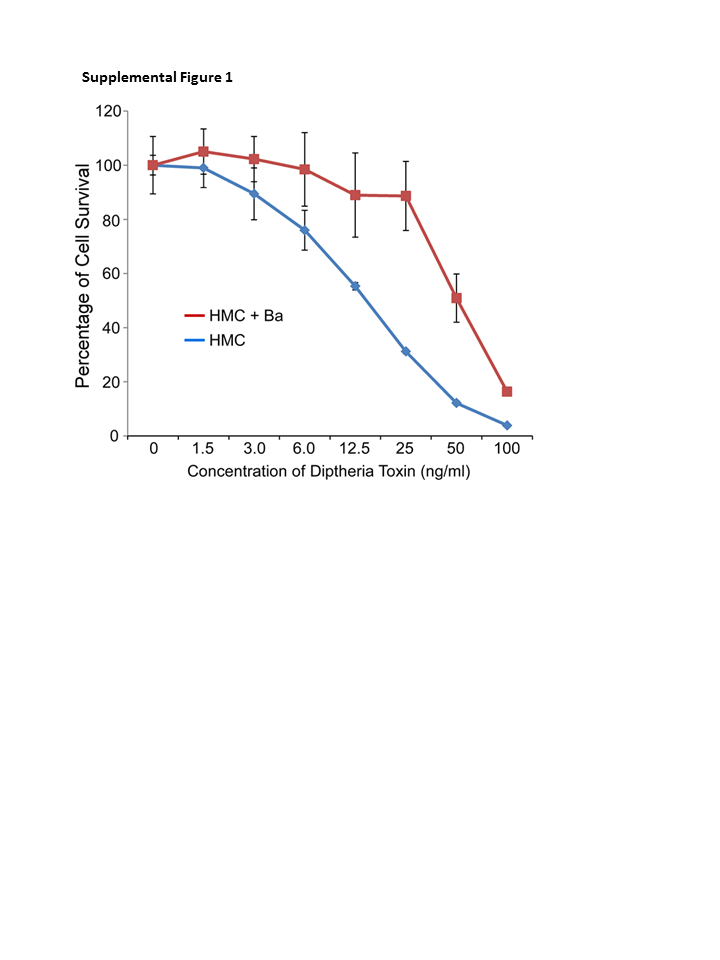

Supplement: Figure S1 — A. Bacitracin inhibits PDI-dependent DT toxicity. HMCs were treated with DT with or without bacitracin. Bacitracin increased cell survival significantly, demonstrating the ability to inhibit PDI-dependent cell toxicity (*p<0.05). (Error bars, +SD from 3 independent experiments). (TIF) [file pone.0112986.s001.tif]

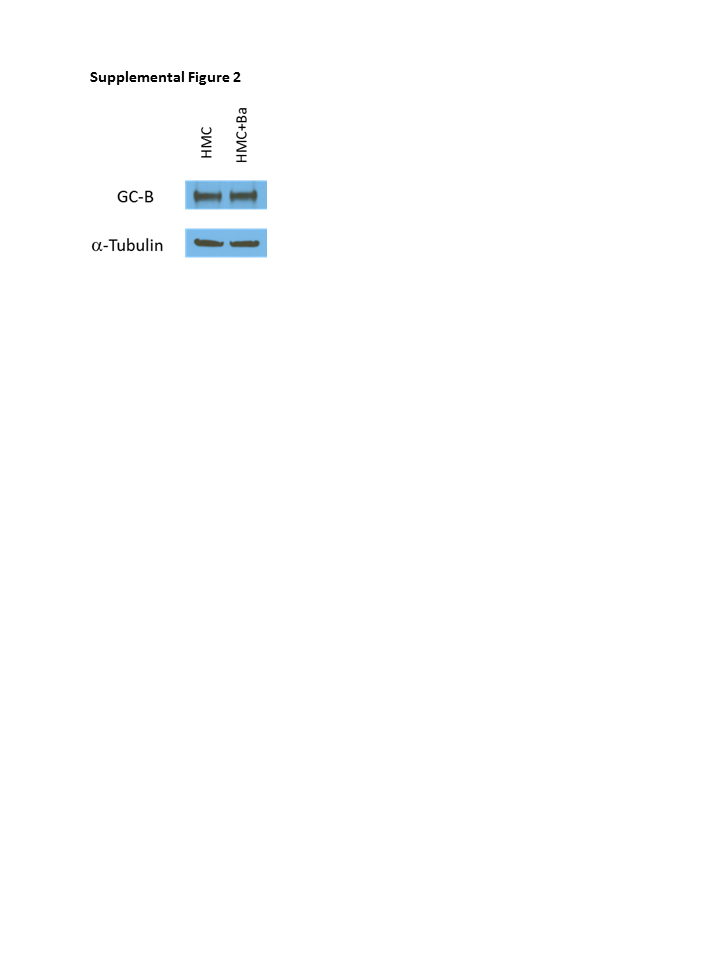

Supplement: Figure S2 — Bacitracin treatment does not affect GC-B expression on HMC. Western blot analysis of HMCs with and without Bacitracin treatment. There was no difference in GC-B expression with exposure to Ba. (TIF) [file pone.0112986.s002.tif]
